# Supplementary material for: Informed forecasting: Leveraging auxiliary knowledge to boost large language model performance on time series forecasting
Source: PLoS One. 2026 Oct 1;21(10):e0358970. doi: 10.1371/journal.pone.0358970 (PMC13630261; doi:10.1371/journal.pone.0358970)
Supplement: Supplementary file 1 [file pone.0358970.s002.pdf]

Response to Referees, PONE-D-26-08442: Informed Forecasting: Leveraging Auxiliary Knowledge to Boost Large Language Models Performance on Time Series Forecasting

---

We thank the editorial team and the reviewers for providing constructive feedback on our manuscript. We believe that addressing these comments has contributed significantly to the quality of the paper. Enclosed with this letter, please find our responses to the constructive feedback from the referees as well as the new version of the manuscript. In what follows, we provide both the comments of the referees (in black), and our responses (in pink). The manuscript also highlights changes in blue. If additional information would be helpful, please do not hesitate to contact us.

1. Reviewer 1

This manuscript presents a well-designed empirical study on enhancing large language models (LLMs) for time series forecasting via time-dependent covariates. The experimental framework is rigorous, utilizing diverse real-world datasets, adequate baseline comparisons, and robust statistical validations, making the methodology technically sound. However, a few critical issues regarding reference accuracy must be addressed prior to publication.

- Reference 9: The citation for the "PromptCast" paper is incomplete and lists an incorrect year. Please update it to the official publication.

  We thank the reviewer for pointing this out. We have updated Reference 9 to cite the official publication of the PromptCast paper and corrected the year accordingly.

- References 27 and 33: These entries are duplicates of the same paper by Gruver et al. Please remove the redundancy and consolidate the in-text citations.

  We thank the reviewer for identifying this duplication. We have removed the redundant entry and consolidated the corresponding in-text citations in the revised manuscript.

- References 30 and 34: Reference 30 cites the arXiv preprint of TimeCMA, while Reference 34 cites the officially accepted AAAI 2025 version. Please remove Reference 30 and direct all in-text citations to the published conference version (Reference 34). We thank the reviewer for this helpful correction.

  We have removed the arXiv preprint citation and updated all relevant in-text citations to refer to the officially accepted AAAI 2025 version of TimeCMA.

2. Reviewer 2

This study investigates how time-dependent covariates can be integrated into prompt design for large language models to improve time series forecasting performance across multiple real-world datasets and forecasting settings. The manuscript needs a major revision to be accepted.

- The abstract clearly states the conceptual goal, but it does not identify which LLMs, baseline forecasting methods, or evaluation metrics were used; including this information would make the reported improvements more interpretable and technically credible.

- Although the abstract claims statistically significant improvements, it does not provide any representative quantitative results or effect sizes; adding concise numerical evidence would strengthen the impact of the contribution.

  We thank the reviewer for raising these two important point regarding the abstract. We have revised the abstract to specify the LLM used in our experiments and to include a representative quantitative improvement in forecasting accuracy. This revision provides readers with a clearer initial understanding of the experimental setting and the magnitude of the observed error reduction.

- The introduction should clearly conclude with a distinct section highlighting the novel contributions of your work.

  We thank the reviewer for this helpful suggestion. We have revised the end of the Introduction to include a distinct paragraph that clearly summarizes the novel contributions of the paper. Specifically, the revised paragraph highlights three main contributions: the proposed covariate-informed prompting framework for LLM-based time series forecasting, the comparison of representative prompt formats for organizing covariate information, and the validation-based procedure for selecting prompt–covariate pairs for a given dataset and forecast horizon.

- At the ending of the intro, it is advised to add a para that mentions briefly what each next section contains.

  We thank the reviewer for this helpful suggestion. We agree that adding a brief roadmap at the end of the Introduction improves the organization and readability of the manuscript. Accordingly, we have added a paragraph summarizing the contents of the following sections.

- The literature review should benefit from more explorations of previous studies.

  We thank the reviewer for this helpful suggestion. We have expanded the literature review by adding recent studies that provide broader context on LLM-based time series analysis and foundation models for time series forecasting. Specifically, we now cite recent survey papers that discuss emerging directions in this area, including prompt-based forecasting, numerical tokenization, representation alignment, multimodal integration, and tool-augmented forecasting. We also added a recent study on large pretrained transformer models for time series, which further highlights the rapid development of foundation-model approaches in temporal analysis. These additions strengthen the positioning of our work within the broader literature while keeping the focus of the manuscript on covariate-informed prompting for LLM-based forecasting.

- The discussion section needs to be expanded to more thoroughly analyze the results. We thank the reviwer for the helpful comment. We have now added a discussion in Sections 4 and 5 to further clarify the results.

- The first paragraph of the conclusion should succinctly summarize the contributions of the study in past tense. We thank the reviewer for this helpful comment. We have revised the first paragraph of the Conclusion to succinctly summarize the study's contributions in the past tense.

- The second paragraph of the conclusion should provide clear and actionable future recommendations. We thank the reviewer for the helpful comment. We have revised the second paragraph of the Conclusion to include clearer and more actionable future recommendations. Specifically, the revised paragraph now outlines two concrete directions: (1) extending the framework to probabilistic forecasting by prompting for calibrated quantiles, prediction intervals, or full predictive distributions, evaluated with proper scoring rules and empirical coverage; and (2) generalizing the method to multivariate settings by conditioning on multiple synchronized series and cross-series covariates, building on approaches such as TimeCMA [4].

- Equations are not properly cited, please add original references. We thank the reviewer for this helpful comment. We have added an appropriate reference for the evaluation metrics used in the study, including MAE, MAPE, and RMSE [2].

- Merge the related sections please. We thank the reviewer for the helpful comment. We have now merged previous Sections 2 (Time Series Forecasting) and 3 (LLMs as Time Series Forecasting Models) into Section 2 (Related Work). Also We have merged Sections 5 (Experiments Setting) and 6(Numerical Results) into Section 4 (Numerical Experiments) and have merged related subsections.

3. Reviewer 3

This paper proposes a prompt engineering pipeline for LLM-based time series forecasting that incorporates time-dependent covariates, evaluated on three public datasets using GPT-4o-mini.

- The entire study relies on a single proprietary model, GPT-4o-mini. It is unclear whether the observed benefits of covariate integration are specific to GPT-4o-mini's pretraining data and tokenization or whether they hold for other LLMs such as open-source models (LLaMA-3, Mistral, or Gemma). Without at least one additional model, the results cannot be claimed as general findings about LLM-based forecasting; they describe GPT-4o-mini behavior on these three datasets.

  We thank the reviewer for this helpful comment. We agree that, because the experiments in the present study are conducted using a single proprietary model, GPT-4o-mini, the results should not be interpreted as model-independent conclusions about all LLMs. We have revised the manuscript to clarify this scope and now state that the empirical findings describe the behavior of GPT-4o-mini on the three datasets considered in this study. We also acknowledge that the effectiveness of covariate integration may depend on the model architecture, pretraining data, tokenization scheme, instruction-following behavior, and numerical reasoning capability.

  The objective of this paper is not to claim that including covariates is guaranteed to improve the forecasting performance of every LLM. Rather, our goal is to highlight that, when an LLM is used for time series forecasting, horizon-known covariates can be considered as a practical source of auxiliary information. Since the benefit of covariate integration is problem- and model-dependent, we propose a validation-based procedure to evaluate whether the additional covariate information improves forecasting accuracy for a given dataset, model, and forecast horizon. We have also added a reference to our related work [1], where the effect of covariate integration is discussed for additional LLM variants.

- The "Coupled" prompt is identified as the best format across nearly all settings, yet the paper offers no theoretical or mechanistic explanation for why this is the case. The attention-based intuition offered in Section 4.2 is speculative and not supported by any attention analysis or ablation. A brief experiment examining why decoupled and contextualized formats underperform, for example by varying prompt length or testing with and without structural delimiters, would strengthen this central claim.

  We thank the reviewer for this important comment. We agree that our previous attention-based explanation was speculative because we did not perform an attention analysis or an ablation study on prompt length, delimiters, or structural formatting. To address this concern, we have revised the manuscript to avoid presenting the Coupled prompt's superior performance as a mechanistic or theoretical conclusion.

  In the revised manuscript, we now frame the Coupled prompt's advantage as an empirical observation from our experiments. We provide a more cautious interpretation based on prompt structure: the Coupled format places each covariate value immediately next to its corresponding target value, thereby preserving local alignment between the historical observations and their temporal descriptors. This may make the forecasting task easier for the model because the future covariates are presented in the same key-value format, with the target values left to be predicted. In contrast, the Decoupled and Contextualized formats separate the covariate and target sequences, which may require the model to reconstruct their alignment before forecasting. The Contextualized format also introduces additional natural-language tokens, which may increase prompt length without necessarily improving numerical prediction.

  We have also explicitly acknowledged that this explanation is not supported by direct attention analysis and that we do not claim the Coupled prompt is theoretically optimal. We added a limitation noting

that future work should examine prompt-length effects, delimiter choices, structural alignment, and attention patterns to better explain why different prompt formats perform differently.

- The validation-based selection procedure requires running inference for every (prompt format, covariate) pair on the validation set. With 3 prompt types and 3 covariate options per dataset, this is 9 API calls per prediction step, and the paper acknowledges that covariate inclusion roughly doubles token count. The reported cost in dollars and wall-clock time is absent. Given that PLOS ONE serves a broad audience including practitioners without large API budgets, a cost table or at least an order-of-magnitude estimate is needed.

  We have now added a discussion in Section 4. Specifically, we explain that the computational cost of the algorithm is low, with the entire prediction process taking only on the order of seconds. We also clarify that the monetary cost of prediction depends on the choice of model. For the model used in this paper, GPT-4o Mini, this cost is very low; for the prediction task in Section 4, the cost is less than $0.01.

- All three covariates used, year, month, date, and day-of-week, are calendar attributes that are trivially available in any time series. The paper claims to focus on "covariates that are not tied to a specific dataset," but this design choice also limits the contribution. Calendar features are already internally represented in large pretrained models, so it is not clear whether the gains come from the prompt engineering format or simply from reminding the model of a date it could infer from context. An experiment comparing explicit date covariates versus implicit cues (e.g., telling the model "this is week 48 of the year" versus providing the date string) would clarify this. We thank the reviewer for this insightful comment. We agree that the use of calendar-based covariates, such as year, month, date, and day of week, limits the scope of the conclusions that can be drawn from the present experiments. Our intention was to focus on covariates that are widely available, known in advance for the forecast horizon, and not tied to a specific application domain. We have revised the manuscript to clarify this point and to avoid overclaiming the generality of the results.

  Specifically, we now explain that the observed improvements should be interpreted as evidence that explicitly aligning historical observations and future prediction points with simple calendar covariates can improve prompt-based LLM forecasting. We also clarify that, although pretrained LLMs may contain general calendar knowledge, the model does not automatically know that the provided values should be interpreted as calendar-based covariates aligned with the target series. Without explicit prompt structure, these values may be treated merely as additional tokens or labels rather than as temporally meaningful variables for forecasting. Thus, the covariates in our framework act not only as calendar reminders but also as structured anchors that make the relationship between past observations and future forecast points visible to the model.

  At the same time, we acknowledge that our current experiments do not disentangle whether the improvement is due to the semantic meaning of the calendar variables, the explicit prompt structure, or the interaction between the two. We have therefore added this point as a limitation and identified the comparison between explicit calendar covariates and implicit temporal cues as an important direction for future work.

- The following references are recommended to consider to strengthen the manuscript's discussion. [1] https://doi.org/10.1109/TPEL.2025.3618228 [2] https://doi.org/10.1109/TIE.2026.3658639

  We thank the reviewer for these recommendations. We have read both papers and incorporated them into the Introduction. Specifically, we have added a sentence to the paragraph that discusses application domains of time series forecasting, noting that data-driven models have been applied to fault diagnosis and stability assessment in power systems and industrial operations [3, 5]. This addition broadens the scope of motivating applications presented in the Introduction and provides appropriate credit to relevant prior work in these domains.

References

[1] Ghasemloo, M. and Moradi, A. (2025). Forecasting the future: Has gpt-5 improved time series forecasting accuracy over gpt-4? *Available at SSRN 5402179*.

[2] Hyndman, R. J. and Koehler, A. B. (2006). Another look at measures of forecast accuracy. *International journal of forecasting*, 22(4):679–688.

[3] Lin, H., Chung, H. S.-H., Lin, C., Xie, D., Deng, Q., Lyu, M., Goetz, S. M., Chen, J., and Ge, X. (2025). Improved fault diagnosis capability in chbmcs: Counter design for multiple oc switches via an e-svm unit. *IEEE Transactions on Power Electronics*.

[4] Liu, C., Xu, Q., Miao, H., Yang, S., Zhang, L., Long, C., Li, Z., and Zhao, R. (2025). Timecma: Towards llm-empowered multivariate time series forecasting via cross-modality alignment. In *Proceedings of the AAAI Conference on Artificial Intelligence*, volume 39, pages 18780–18788.

[5] Meng, X., Xie, D., Lin, H., Lin, C., Ge, X., and Liu, Z. (2026). Dissipativity-based multiport stability root-cause identification and mitigation for solid-state transformers. *IEEE Transactions on Industrial Electronics*.
