# Supplementary material for: Informed forecasting: Leveraging auxiliary knowledge to boost large language model performance on time series forecasting
Source: PLoS One. 2026 Oct 1;21(10):e0358970. doi: 10.1371/journal.pone.0358970 (PMC13630261; doi:10.1371/journal.pone.0358970)
Supplement: Supplementary file 2 [file pone.0358970.s003.pdf]

Response to Referees, PONE-D-26-08442R1: *Informed Forecasting: Leveraging Auxiliary Knowledge to Boost Large Language Model Performance on Time Series Forecasting*

We sincerely thank the Academic Editor and Reviewer #2 for their careful evaluation of our revised manuscript. We are grateful that Reviewer #2 confirmed that all comments from the previous round had been addressed. We also appreciate the request for a final minor English proofreading pass.

In response, we carefully proofread the entire manuscript and made several minor corrections throughout the paper. All changes made in this revision are highlighted in blue in the marked-up manuscript. These revisions are editorial in nature and do not alter the methodology, experimental results, statistical analysis, or scientific conclusions of the paper.

1. Reviewer 2

Reviewer comment:

"All comments have been addressed."

We sincerely thank the reviewer for confirming that the comments raised in the previous round have been adequately addressed. We greatly appreciate the reviewer's careful reading of the manuscript and the constructive feedback provided throughout the review process.

Reviewer comment:

"Well responded, now kindly conduct minor English proofreading for the manuscript as there are some typos."

We thank the reviewer for this helpful final comment. We have carefully proofread the complete manuscript and made several minor corrections to improve the English presentation. The revisions include corrections to grammar, typographical errors, punctuation, wording, article usage, sentence structure, capitalization, terminology consistency, and minor cross-references. We also corrected one minor description of the Call Center evaluation period to ensure consistency with the stated validation and testing split.

We sincerely thank the Academic Editor and the reviewer for their time and consideration of our manuscript.
