# Supplementary material for: Informed forecasting: Leveraging auxiliary knowledge to boost large language model performance on time series forecasting
Source: PLoS One. 2026 Oct 1;21(10):e0358970. doi: 10.1371/journal.pone.0358970 (PMC13630261; doi:10.1371/journal.pone.0358970)
Supplement: Peer review history

# Peer review: Decision Letter 0

**DOI:** 10.1371/journal.pone.0358970.r001

**Authors:** Dinesh Nishad

18 Apr 2026

Dear Dr. Ghasemloo,

Thank you for submitting your manuscript to PLOS ONE. After careful consideration, we feel that it has merit but does not fully meet PLOS ONE’s publication criteria as it currently stands. Therefore, we invite you to submit a revised version of the manuscript that addresses the points raised during the review process.

Please submit your revised manuscript by Jun 02 2026 11:59PM. If you will need more time than this to complete your revisions, please reply to this message or contact the journal office at plosone@plos.org. When you're ready to submit your revision, log on to https://www.editorialmanager.com/pone/ and select the 'Submissions Needing Revision' folder to locate your manuscript file.

- A letter that responds to each point raised by the academic editor and reviewer(s). You should upload this letter as a separate file labeled 'Response to Reviewers'.
- A marked-up copy of your manuscript that highlights changes made to the original version. You should upload this as a separate file labeled 'Revised Manuscript with Track Changes'.
- An unmarked version of your revised paper without tracked changes. You should upload this as a separate file labeled 'Manuscript'.

If you would like to make changes to your financial disclosure, please include your updated statement in your cover letter. Guidelines for resubmitting your figure files are available below the reviewer comments at the end of this letter.

If applicable, we recommend that you deposit your laboratory protocols in protocols.io to enhance the reproducibility of your results. Protocols.io assigns your protocol its own identifier (DOI) so that it can be cited independently in the future. For instructions see: https://journals.plos.org/plosone/s/submission-guidelines#loc-laboratory-protocols. Additionally, PLOS ONE offers an option for publishing peer-reviewed Lab Protocol articles, which describe protocols hosted on protocols.io. Read more information on sharing protocols at https://plos.org/protocols?utm_medium=editorial-email&utm_source=authorletters&utm_campaign=protocols.

As the corresponding author, your ORCID iD is verified in the submission system and will appear in the published article. PLOS supports the use of ORCID, and we encourage all coauthors to register for an ORCID iD and use it as well. Please encourage your coauthors to verify their ORCID iD within the submission system before final acceptance, as unverified ORCID iDs will not appear in the published article. *Only*  the individual author can complete the verification step; PLOS staff *cannot*  verify ORCID iDs on behalf of authors.

We look forward to receiving your revised manuscript.

Kind regards,

Dinesh Kumar Nishad, PhD

Academic Editor

PLOS One

Journal Requirements:

When submitting your revision, we need you to address these additional requirements.

1.Please ensure that your manuscript meets PLOS ONE's style requirements, including those for file naming. The PLOS ONE style templates can be found at

https://journals.plos.org/plosone/s/file?id=wjVg/PLOSOne_formatting_sample_main_body.pdf and

https://journals.plos.org/plosone/s/file?id=ba62/PLOSOne_formatting_sample_title_authors_affiliations.pdf

2. PLOS requires an ORCID iD for the corresponding author in Editorial Manager on papers submitted after December 6th, 2016. Please ensure that you have an ORCID iD and that it is validated in Editorial Manager. To do this, go to ‘Update my Information’ (in the upper left-hand corner of the main menu), and click on the Fetch/Validate link next to the ORCID field. This will take you to the ORCID site and allow you to create a new iD or authenticate a pre-existing iD in Editorial Manager.

3. Please note that PLOS One has specific guidelines on code sharing for submissions in which author-generated code underpins the findings in the manuscript. In these cases, we expect all author-generated code to be made available without restrictions upon publication of the work. Please review our guidelines at https://journals.plos.org/plosone/s/materials-and-software-sharing#loc-sharing-code and ensure that your code is shared in a way that follows best practice and facilitates reproducibility and reuse.

4. Please note that your Data Availability Statement is currently missing the repository name and/or the DOI/accession number of each dataset OR a direct link to access each database. If your manuscript is accepted for publication, you will be asked to provide these details on a very short timeline. We therefore suggest that you provide this information now, though we will not hold up the peer review process if you are unable.

5. If the reviewer comments include a recommendation to cite specific previously published works, please review and evaluate these publications to determine whether they are relevant and should be cited. There is no requirement to cite these works unless the editor has indicated otherwise.

[Note: HTML markup is below. Please do not edit.]

Reviewer's Responses to Questions

**Comments to the Author**

1. Is the manuscript technically sound, and do the data support the conclusions?

Reviewer #1: Yes

Reviewer #2: Partly

Reviewer #3: Yes

**********

2. Has the statistical analysis been performed appropriately and rigorously?

Reviewer #1: Yes

Reviewer #2: N/A

Reviewer #3: N/A

**********

3. Have the authors made all data underlying the findings in their manuscript fully available?

The PLOS Data policy

Reviewer #1: Yes

Reviewer #2: Yes

Reviewer #3: Yes

**********

4. Is the manuscript presented in an intelligible fashion and written in standard English?

Reviewer #1: Yes

Reviewer #2: Yes

Reviewer #3: Yes

**********

Reviewer #1: This manuscript presents a well-designed empirical study on enhancing large language models (LLMs) for time series forecasting via time-dependent covariates. The experimental framework is rigorous, utilizing diverse real-world datasets, adequate baseline comparisons, and robust statistical validations, making the methodology technically sound. However, a few critical issues regarding reference accuracy must be addressed prior to publication.

1. Reference 9: The citation for the "PromptCast" paper is incomplete and lists an incorrect year. Please update it to the official publication.

2. References 27 and 33: These entries are duplicates of the same paper by Gruver et al. Please remove the redundancy and consolidate the in-text citations.

3. References 30 and 34: Reference 30 cites the arXiv preprint of TimeCMA, while Reference 34 cites the officially accepted AAAI 2025 version. Please remove Reference 30 and direct all in-text citations to the published conference version (Reference 34).

Reviewer #2: This study investigates how time-dependent covariates can be integrated into prompt design for large language models to improve time series forecasting performance across multiple real-world datasets and forecasting settings. The manuscript needs a major revision to be accepted.

1. The abstract clearly states the conceptual goal, but it does not identify which LLMs, baseline forecasting methods, or evaluation metrics were used; including this information would make the reported improvements more interpretable and technically credible.

2. Although the abstract claims statistically significant improvements, it does not provide any representative quantitative results or effect sizes; adding concise numerical evidence would strengthen the impact of the contribution.

3. The introduction should clearly conclude with a distinct section highlighting the novel contributions of your work.

4. At the ending of the intro, it is advised to add a para that mentions briefly what each next section contains.

5. The literature review should benefit from more explorations of previous studies.

6. The discussion section needs to be expanded to more thoroughly analyze the results.

7. The first paragraph of the conclusion should succinctly summarize the contributions of the study in past tense.

8. The second paragraph of the conclusion should provide clear and actionable future recommendations.

9. Equations are not properly cited, please add original references.

10. Merge the related sections please.

Reviewer #3: This paper proposes a prompt engineering pipeline for LLM-based time series forecasting that incorporates time-dependent covariates, evaluated on three public datasets using GPT-4o-mini.

1. The entire study relies on a single proprietary model, GPT-4o-mini. It is unclear whether the observed benefits of covariate integration are specific to GPT-4o-mini's pretraining data and tokenization or whether they hold for other LLMs such as open-source models (LLaMA-3, Mistral, or Gemma). Without at least one additional model, the results cannot be claimed as general findings about LLM-based forecasting; they describe GPT-4o-mini behavior on these three datasets.

2. The "Coupled" prompt is identified as the best format across nearly all settings, yet the paper offers no theoretical or mechanistic explanation for why this is the case. The attention-based intuition offered in Section 4.2 is speculative and not supported by any attention analysis or ablation. A brief experiment examining why decoupled and contextualized formats underperform, for example by varying prompt length or testing with and without structural delimiters, would strengthen this central claim.

3. The validation-based selection procedure requires running inference for every (prompt format, covariate) pair on the validation set. With 3 prompt types and 3 covariate options per dataset, this is 9 API calls per prediction step, and the paper acknowledges that covariate inclusion roughly doubles token count. The reported cost in dollars and wall-clock time is absent. Given that PLOS ONE serves a broad audience including practitioners without large API budgets, a cost table or at least an order-of-magnitude estimate is needed.

4. All three covariates used, year, month, date, and day-of-week, are calendar attributes that are trivially available in any time series. The paper claims to focus on "covariates that are not tied to a specific dataset," but this design choice also limits the contribution. Calendar features are already internally represented in large pretrained models, so it is not clear whether the gains come from the prompt engineering format or simply from reminding the model of a date it could infer from context. An experiment comparing explicit date covariates versus implicit cues (e.g., telling the model "this is week 48 of the year" versus providing the date string) would clarify this.

5. The following references are recommended to consider to strengthen the manuscript's discussion.

[1] https://doi.org/10.1109/TPEL.2025.3618228

[2] https://doi.org/10.1109/TIE.2026.3658639

**********

what does this mean?). If published, this will include your full peer review and any attached files.

If you choose “no”, your identity will remain anonymous but your review may still be made public.

**Do you want your identity to be public for this peer review?**  For information about this choice, including consent withdrawal, please see our Privacy Policy

Reviewer #1: No

Reviewer #2: No

Reviewer #3: No

**********

[NOTE: If reviewer comments were submitted as an attachment file, they will be attached to this email and accessible via the submission site. Please log into your account, locate the manuscript record, and check for the action link "View Attachments". If this link does not appear, there are no attachment files.]

To ensure your figures meet our technical requirements, please review our figure guidelines: https://journals.plos.org/plosone/s/figures

You may also use PLOS’s free figure tool, NAAS, to help you prepare publication quality figures: https://journals.plos.org/plosone/s/figures#loc-tools-for-figure-preparation.

NAAS will assess whether your figures meet our technical requirements by comparing each figure against our figure specifications.

# Author comment: Author response to Decision Letter 1

**DOI:** 10.1371/journal.pone.0358970.r002

27 Jun 2026

We thank the editor and reviewers for their helpful and constructive comments. We have made every effort to address the comments carefully and believe that the revisions have significantly strengthened the manuscript. For detailed point-by-point responses, please refer to the accompanying Response to Reviewers file. If any additional revisions or clarifications are needed, we would be happy to provide them.

Attachment Submitted filename: Response to Reviewers.pdf

# Peer review: Decision Letter 1

**DOI:** 10.1371/journal.pone.0358970.r003

**Authors:** Dinesh Nishad

18 Aug 2026

Dear Dr. Ghasemloo,

Thank you for submitting your manuscript to PLOS One. After careful consideration, we feel that it has merit but does not fully meet PLOS One’s publication criteria as it currently stands. Therefore, we invite you to submit a revised version of the manuscript that addresses the points raised during the review process.

Please submit your revised manuscript by Oct 02 2026 11:59PM. If you will need more time than this to complete your revisions, please reply to this message or contact the journal office at plosone@plos.org. When you're ready to submit your revision, log on to https://www.editorialmanager.com/pone/ and select the 'Submissions Needing Revision' folder to locate your manuscript file.

- A letter that responds to each point raised by the academic editor and reviewer(s). You should upload this letter as a separate file labeled 'Response to Reviewers'.
- A marked-up copy of your manuscript that highlights changes made to the original version. You should upload this as a separate file labeled 'Revised Manuscript with Track Changes'.
- An unmarked version of your revised paper without tracked changes. You should upload this as a separate file labeled 'Manuscript'.

If you would like to make changes to your financial disclosure, please include your updated statement in your cover letter. Guidelines for resubmitting your figure files are available below the reviewer comments at the end of this letter.

If applicable, we recommend that you deposit your laboratory protocols in protocols.io to enhance the reproducibility of your results. Protocols.io assigns your protocol its own identifier (DOI) so that it can be cited independently in the future. For instructions see: https://journals.plos.org/plosone/s/submission-guidelines#loc-laboratory-protocols. Additionally, PLOS One offers an option for publishing peer-reviewed Lab Protocol articles, which describe protocols hosted on protocols.io. Read more information on sharing protocols at https://plos.org/protocols?utm_medium=editorial-email&utm_source=authorletters&utm_campaign=protocols.

As the corresponding author, your ORCID iD is verified in the submission system and will appear in the published article. PLOS supports the use of ORCID, and we encourage all coauthors to register for an ORCID iD and use it as well. Please encourage your coauthors to verify their ORCID iD within the submission system before final acceptance, as unverified ORCID iDs will not appear in the published article. *Only*  the individual author can complete the verification step; PLOS staff *cannot*  verify ORCID iDs on behalf of authors.

We look forward to receiving your revised manuscript.

Kind regards,

Dinesh Kumar Nishad, PhD

Academic Editor

PLOS One

Journal Requirements:

If the reviewer comments include a recommendation to cite specific previously published works, please review and evaluate these publications to determine whether they are relevant and should be cited. There is no requirement to cite these works unless the editor has indicated otherwise.

Please review your reference list to ensure that it is complete and correct. If you have cited papers that have been retracted, please include the rationale for doing so in the manuscript text, or remove these references and replace them with relevant current references. Any changes to the reference list should be mentioned in the rebuttal letter that accompanies your revised manuscript. If you need to cite a retracted article, indicate the article’s retracted status in the References list and also include a citation and full reference for the retraction notice.

[Note: HTML markup is below. Please do not edit.]

Reviewers' comments:

Reviewer's Responses to Questions

**Comments to the Author**

Reviewer #2: All comments have been addressed

**********

2. Is the manuscript technically sound, and do the data support the conclusions?

Reviewer #2: (No Response)

**********

3. Has the statistical analysis been performed appropriately and rigorously?

Reviewer #2: (No Response)

**********

4. Have the authors made all data underlying the findings in their manuscript fully available?

The PLOS Data policy

Reviewer #2: (No Response)

**********

5. Is the manuscript presented in an intelligible fashion and written in standard English?

Reviewer #2: (No Response)

**********

Reviewer #2: Well responded, now kindly conduct minor English proofreading for the manuscript as there are some typos.

**********

what does this mean?). If published, this will include your full peer review and any attached files.

If you choose “no”, your identity will remain anonymous but your review may still be made public.

**Do you want your identity to be public for this peer review?**  For information about this choice, including consent withdrawal, please see our Privacy Policy

Reviewer #2: No

**********

[NOTE: If reviewer comments were submitted as an attachment file, they will be attached to this email and accessible via the submission site. Please log into your account, locate the manuscript record, and check for the action link "View Attachments". If this link does not appear, there are no attachment files.]

To ensure your figures meet our technical requirements, please review our figure guidelines: https://journals.plos.org/plosone/s/figures

You may also use PLOS’s free figure tool, NAAS, to help you prepare publication quality figures: https://journals.plos.org/plosone/s/figures#loc-tools-for-figure-preparation.

NAAS will assess whether your figures meet our technical requirements by comparing each figure against our figure specifications.

# Author comment: Author response to Decision Letter 2

**DOI:** 10.1371/journal.pone.0358970.r004

26 Aug 2026

We sincerely thank the Academic Editor and Reviewer 2 for their careful evaluation of our revised manuscript. We are grateful that Reviewer 2 confirmed that all comments from the previous round had been addressed. We also appreciate the request for a final minor English proofreading pass.

In response, we carefully proofread the entire manuscript and made several minor corrections throughout the paper.

All changes made in this revision are highlighted in blue in the marked-up manuscript. These revisions are editorial in nature and do not alter the methodology, experimental results, statistical analysis, or scientific conclusions of the paper.

Attachment Submitted filename: Response to Reviews Minor Revision.pdf

# Peer review: Decision Letter 2

**DOI:** 10.1371/journal.pone.0358970.r005

**Authors:** Dinesh Nishad

8 Sep 2026

Informed forecasting: Leveraging auxiliary knowledge to boost large language model performance on time series forecasting

PONE-D-26-08442R2

Dear Dr. Ghasemloo,

We’re pleased to inform you that your manuscript has been judged scientifically suitable for publication and will be formally accepted for publication once it meets all outstanding technical requirements.

Within one week, you’ll receive an e-mail detailing the required amendments. When these have been addressed, you’ll receive a formal acceptance letter and your manuscript will be scheduled for publication.

An invoice will be generated when your article is formally accepted. Please note, if your institution has a publishing partnership with PLOS and your article meets the relevant criteria, all or part of your publication costs will be covered. Please make sure your user information is up-to-date by logging into Editorial Manager at Editorial Manager® and clicking the ‘Update My Information' link at the top of the page. For questions related to billing, please contact billing support.

If your institution or institutions have a press office, please notify them about your upcoming paper to help maximize its impact. If they’ll be preparing press materials, please inform our press team as soon as possible -- no later than 48 hours after receiving the formal acceptance. Your manuscript will remain under strict press embargo until 2 pm Eastern Time on the date of publication. For more information, please contact onepress@plos.org.

Kind regards,

Dinesh Kumar Nishad, PhD

Academic Editor

PLOS One

Additional Editor Comments (optional):

Reviewers' comments:

Reviewer's Responses to Questions

**Comments to the Author**

Reviewer #2: (No Response)

**********

2. Is the manuscript technically sound, and do the data support the conclusions?

Reviewer #2: (No Response)

**********

3. Has the statistical analysis been performed appropriately and rigorously?

Reviewer #2: (No Response)

**********

4. Have the authors made all data underlying the findings in their manuscript fully available?

The PLOS Data policy

Reviewer #2: (No Response)

**********

5. Is the manuscript presented in an intelligible fashion and written in standard English?

Reviewer #2: (No Response)

**********

Reviewer #2: (No Response)

**********

what does this mean?). If published, this will include your full peer review and any attached files.

If you choose “no”, your identity will remain anonymous but your review may still be made public.

**Do you want your identity to be public for this peer review?**  For information about this choice, including consent withdrawal, please see our Privacy Policy

Reviewer #2: No

**********

# Editor report: Acceptance letter

**DOI:** 10.1371/journal.pone.0358970.r006

**Authors:** Dinesh Nishad

PONE-D-26-08442R2

PLOS One

Dear Dr. Ghasemloo,

I'm pleased to inform you that your manuscript has been deemed suitable for publication in PLOS One. Congratulations! Your manuscript is now being handed over to our production team.

At this stage, our production department will prepare your paper for publication. This includes ensuring the following:

* All references, tables, and figures are properly cited

* All relevant supporting information is included in the manuscript submission,

* There are no issues that prevent the paper from being properly typeset

You will receive further instructions from the production team, including instructions on how to review your proof when it is ready. Please keep in mind that we are working through a large volume of accepted articles, so please give us a few days to review your paper and let you know the next and final steps.

Lastly, if your institution or institutions have a press office, please let them know about your upcoming paper now to help maximize its impact. If they'll be preparing press materials, please inform our press team within the next 48 hours. Your manuscript will remain under strict press embargo until 2 pm Eastern Time on the date of publication. For more information, please contact onepress@plos.org.

You will receive an invoice from PLOS for your publication fee after your manuscript has reached the completed accept phase. If you receive an email requesting payment before acceptance or for any other service, this may be a phishing scheme. Learn how to identify phishing emails and protect your accounts at https://explore.plos.org/phishing.

If we can help with anything else, please email us at customercare@plos.org.

Thank you for submitting your work to PLOS One and supporting open access.

Kind regards,

PLOS One Editorial Office Staff

on behalf of

Dr. Dinesh Kumar Nishad

Academic Editor

PLOS One
